# Incremental value of early systolic lengthening and postsystolic shortening in detecting left anterior descending artery stenosis using nonstress speckle-tracking echocardiography

**DOI:** 10.1038/s41598-021-98900-1

**Published:** 2021-09-29

**Authors:** Takako Ishigaki, Toshihiko Asanuma, Noriaki Yagi, Hiromi Izumi, Shoko Shimizu, Yoshihisa Fujisawa, Toshiyuki Ikemoto, Ryoji Kushima, Kasumi Masuda, Satoshi Nakatani

**Affiliations:** 1 Department of Clinical Laboratory Medicine, Shiga University of Medical Science, Otsu, Shiga Japan; 2 Division of Functional Diagnostics, Department of Health Sciences, Osaka University Graduate School of Medicine, 1-7 Yamadaoka, Suita, Osaka 565-0871 Japan; 3 Department of Cardiovascular Medicine, Shiga University of Medical Science, Otsu, Shiga Japan

**Keywords:** Cardiology, Medical research

## Abstract

The diagnosis of coronary artery disease (CAD) with nonstress echocardiography remains challenging. Although the assessment of either early systolic lengthening (ESL) or postsystolic shortening (PSS) allows the sensitive detection of CAD, it is unclear whether the integrated analysis of ESL and PSS in addition to the peak systolic strain can improve the diagnostic accuracy. We investigated the incremental value of ESL and PSS in detecting left anterior descending artery (LAD) stenosis using nonstress speckle-tracking echocardiography. Fifty-nine patients with significant LAD stenosis but without visual wall motion abnormalities on echocardiography at rest (30 single-vessel stenosis, 29 multivessel stenosis) and 43 patients without significant stenosis of any vessel were enrolled. The peak systolic strain, the time to ESL (T_ESL_), and the time to PSS (T_PSS_) were analyzed in all LAD segments, and the incremental values of the T_ESL_ and T_PSS_ in detecting LAD stenosis and the diagnostic accuracy were evaluated. In the apical anterior segment, the peak systolic strain was significantly lower and T_ESL_ and T_PSS_ were significantly longer in the single-vessel group than in the no stenosis group. In the single-vessel group, the addition of T_ESL_ and T_PSS_ to the peak systolic strain significantly increased the model power in detecting stenosis, and the integrated analysis improved diagnostic accuracy compared with the peak systolic strain alone. In contrast, this incremental value was not demonstrated in the multivessel group. The integrated analysis of the peak systolic strain, ESL, and PSS may allow better screening of single-vessel LAD stenosis using nonstress speckle-tracking echocardiography.

## Introduction

Myocardial ischemia is a pathophysiological state that occurs when myocardial blood flow cannot supply sufficient oxygen for myocardial demands; the imbalance between myocardial oxygen supply and demand is followed by left ventricular (LV) wall motion abnormalities^1^. Stress echocardiography is used to reveal ischemic myocardium in patients with coronary artery disease (CAD). However, the diagnosis of CAD using nonstress echocardiography is still challenging because myocardial ischemia may not occur at rest even in the presence of significant coronary stenosis.

Myocardial strain analysis with tissue Doppler and speckle-tracking echocardiography can evaluate regional myocardial wall motion quantitatively and is useful for identifying subtle abnormal deformations, such as the early systolic lengthening (ESL) and postsystolic shortening (PSS) observed in myocardium with contractile dysfunction^2,3^. Although the assessment of PSS provides the sensitive detection of CAD in stress echocardiography^4,5^ and can offer diagnostic information in nonstress echocardiography^6,7^, the incremental value of PSS over the peak systolic strain in detecting CAD is unclear. Similarly, the assessment of ESL appears to be useful for detecting CAD^8,9^; however, its incremental value is also unknown.

We hypothesized that these subtle deformation abnormalities could emerge even at rest in some patients with significant CAD but without visual wall motion abnormalities and that the analysis of ESL and PSS in addition to the peak systolic strain could allow better screening of CAD in nonstress echocardiography. In this study, we sought to investigate whether the integrated analysis of peak systolic strain, ESL, and PSS could improve the diagnostic accuracy of single-vessel and multivessel left anterior descending artery (LAD) stenosis by using nonstress speckle-tracking echocardiography.

## Methods

### Study population

One hundred thirty-three patients with clinically suspected CAD but without visual wall motion abnormalities on echocardiography at rest between April 2011 and November 2016 were enrolled. In this study, patients with ST elevation or depression on the electrocardiogram were not enrolled because there was a possibility that direct coronary angiography was needed before echocardiography for this population. For all enrolled patients, quantitative coronary angiography (QCA) was performed within 48 h after echocardiography, and the % diameter stenosis was analyzed by one investigator who was blinded to the echocardiographic data. Greater than or equal to 50% diameter stenosis was defined as significant. ESL and PSS can be detected even in the normal myocardium and the incidence is different within the LV segments^10^. For this reason, right coronary artery (RCA) stenosis and circumflex artery (LCX) stenosis were not evaluated in this study to avoid the influence of complicated regional heterogeneity. After excluding patients with single-vessel RCA stenosis, single-vessel LCX stenosis, and two-vessel RCA and LCX stenoses, the remaining patients were divided into three groups: single-vessel stenosis of the LAD (single-vessel group), significant stenoses of ≥ 2 vessels including the LAD (multivessel group), and no significant stenosis in any vessel (no stenosis group). Patients with left ventricular ejection fraction (LVEF) < 50%, atrial fibrillation, frequent arrhythmia, significant valvular disease (more than moderate degree), suboptimal echo images, or ≥ 1 unanalyzable segment in the speckle-tracking analysis were excluded from the study. Finally, 102 patients were included in this study. The study protocol conformed to the ethical principles outlined in the Declaration of Helsinki and was approved by the Shiga University of Medical Science Research Ethics Committee. Written informed consent was acquired from all patients.

### Echocardiography

Two-dimensional echocardiography was performed using Vivid E9 with an M5S-D transducer (GE Healthcare, Horten, Norway) and saved in raw data format. The tissue harmonic mode was used, and the frame rate was set at 52–84 frames/s. LVEF was calculated by the biplane method of disks, and other conventional parameters were also measured according to the recommendations of the American Society of Echocardiography and the European Association of Cardiovascular Imaging, except that early diastolic mitral annular velocity (e′) was obtained only from the septal side^11,12^.

### Speckle-tracking analysis

Speckle-tracking analysis was performed offline using EchoPAC BT11 software (GE Healthcare, Horten, Norway). Apical long-axis, apical 4-chamber, and apical 2-chamber views were captured under breath-hold conditions. End-diastole was defined at the peak of the R wave on the electrocardiogram, and end-systole was defined at the time of aortic valve closure (AVC) determined from the LV outflow velocity. In each apical view, the endocardial border in the end-systolic frame was manually traced, and the wall thickness was set to a width equal to that of the myocardium. The borders were manually adjusted when necessary to optimize the boundary position and tracking. The whole-wall longitudinal strain was then analyzed automatically.

The peak systolic strain, the time to ESL (T_ESL_), and the time to PSS (T_PSS_) were measured from the strain–time curve in one cardiac cycle. T_ESL_ was defined as the time from end-diastole to peak ESL, and T_PSS_ was defined as the time from end-systole (i.e., AVC) to the peak strain during the cardiac cycle. When the peak strain preceded the AVC, T_PSS_ was represented as a negative value. Parameters associated with the amplitude of ESL and PSS were not used because in a preliminary study, we confirmed that the amplitude changes of ESL and PSS were small for patients with significant coronary stenosis but without visual wall motion abnormalities.

The peak systolic strain, T_ESL_, and T_PSS_ were measured in 11 segments perfused by the LAD (apical posterior, apical anteroseptal, mid anteroseptal, basal anteroseptal, mid septal, apical septal, apical lateral, apical inferior, apical anterior, mid anterior, and basal anterior)^11^. The global longitudinal strain (GLS) was also calculated from the 3 apical views. All speckle-tracking analyses were performed by an investigator who was blinded to the QCA data.

In the 11 segments, we first analyzed the diagnostic accuracy of each parameter to differentiate the single-vessel group from the no stenosis group and selected the segment that showed the best diagnostic accuracy in detecting LAD stenosis. Then, in the selected segment, whether the integrated analysis of peak systolic strain, T_ESL_, and T_PSS_ could improve the diagnostic accuracy was evaluated in the single-vessel group and the multivessel group. Furthermore, in patients with successful revascularization confirmed by follow-up QCA, follow-up echocardiography was performed, and the changes in echocardiographic parameters before versus after revascularization were assessed.

### Interobserver and intraobserver reliability

Fifteen image clips were randomly selected from among the total clips to assess interobserver and intraobserver reliability for peak systolic strain, T_ESL_, and T_PSS_ in the apical anterior segment. To determine interobserver reliability, the analysis was repeated by a second observer who was blinded to the values obtained by the first observer. To determine the intraobserver reliability, the analysis was repeated > 2 weeks later by the same observer.

### Statistical analysis

Data are presented as the mean values ± standard deviation or n (%). Normality of the data was assessed with the Shapiro–Wilk test. The comparison among the three groups was performed with the analysis of variance or the Kruskal–Wallis test according to the data normality. For post hoc paired comparisons in normally distributed data, Tukey’s honestly significant difference test or the Games-Howell test was used depending on Levene’s test for equality of variance, whereas Dunn’s test was used in nonnormally distributed data. Pearson’s χ^2^-test or Fisher’s exact test was performed to compare categorical data. The diagnostic accuracy of the single-parameter analysis and the integrated analysis in differentiating the single-vessel or multivessel group from the no stenosis group was evaluated by the area under the receiver operator characteristic (ROC) curve (AUC). The comparison between AUCs was conducted using the method of DeLong et al.^13^. Multivariable logistic regression analysis was performed to establish the independent determinants for the presence of significant LAD stenosis. The independence and incremental value of each measurement were assessed by comparing the model χ^2^ at each step. The comparison of parameters obtained before and after revascularization was performed by the paired *t* test or the Wilcoxon test according to the data normality. Interobserver and intraobserver reliability were determined using intraclass correlation coefficients and Bland–Altman analyses. A *P* value of < 0.05 was defined as statistically significant. The statistical analysis was carried out using IBM SPSS Statistics version 20.0.0 (IBM, Armonk, USA), JMP version 12.0.1, (SAS Institute, Cary, USA), and MedCalc version 19.8 (MedCalc Software, Ostend, Belgium).

## Results

### Patient characteristics

Of the patients included in this study, 59 patients had significant LAD stenosis (30 in the single-vessel group, 29 in the multivessel group), and 43 patients had no significant stenosis of any vessel (no stenosis group). In these patients, the strain–time curves could be obtained in all LV segments. There were no significant differences in clinical and echocardiographic data among the three groups except the GLS. The absolute value of the GLS was significantly lower in the multivessel group than in the no stenosis group. Although three patients in the no stenosis group had positive troponin values (> 0.05 ng/mL), two of them seemed to be false positives because their values were 0.06 ng/mL (Table 1).

**Table 1:** Patient characteristics.

	No stenosis (n = 43)	Single-vessel (n = 30)	Multivessel (n = 29)	P*
Age	69.6 ± 9.8	68.8 ± 8.2	71.5 ± 7.3	0.45
Male	29 (67)	24 (80)	24 (83)	0.26
BSA (m^2^)	1.67 ± 0.20	1.64 ± 0.16	1.66 ± 0.15	0.85
Diabetes	16 (37)	13 (43)	19 (66)	0.55
Hypertension	30 (70)	18 (60)	15 (52)	0.30
Hypercholesterolemia	24 (56)	16 (53)	22 (76)	0.14
Troponin-positive	3(7)	1(3)	3(10)	0.48
LVEDD (mm)	45.4 ± 4.0	45.8 ± 5.5	46.2 ± 3.9	0.78
LVESD (mm)	29.3 ± 3.3	29.5 ± 4.0	30.3 ± 3.0	0.50
LVEF (%)	65.6 ± 3.7	64.8 ± 4.7	63.6 ± 4.0	0.16
LVMI (g/m^2^)	109.1 ± 24.5	108.5 ± 26.2	117.3 ± 21.3	0.28
E/A	0.8 ± 0.2	0.8 ± 0.3	0.8 ± 0.3	0.64
E/e’ (septal)	12.4 ± 3.2	12.0 ± 3.0	13.7 ± 4.0	0.20
GLS (%)	–19.3 ± 2.3	–18.3 ± 2.7	–17.0 ± 3.4†	< 0.01
% diameter stenosis (LAD)	33.3 ± 13.0	61.1 ± 10.2‡	64.9 ± 9.8‡	< 0.01

Data are presented as mean ± standard deviation or n (%).

BSA, body surface area; GLS, global longitudinal strain; LAD, left anterior descending artery; LVEDD, left ventricular end-diastolic dimension; LVEF, left ventricular ejection fraction; LVESD, left ventricular end-systolic dimension; LVMI, left ventricular mass index.

*P derived from the analysis of variance or the Kruskal–Wallis test, †*P* < 0.05 versus no stenosis, ‡*P* < 0.01 versus no stenosis.

### Determination of the optimal segment for detecting LAD stenosis

In differentiating the single-vessel group from the no stenosis group, the apical anterior segment in the apical 2-chamber view demonstrated the largest AUC not only for the peak systolic strain but also for T_ESL_ and T_PSS_. There was no significant difference among the AUCs for the peak systolic strain, T_ESL_ and T_PSS_. In contrast, the apical septal segment in the apical 4-chamber view did not show significant diagnostic accuracy except for T_PSS_ (Table 2). Therefore, the following analyses of speckle-tracking data were performed in the apical anterior segment.

**Table 2:** Receiver operating characteristic curve analysis for differentiating the single-vessel group from the no stenosis group.

	Peak systolic strain		T_ESL_		T_PSS_	
	AUC	*P*	AUC	*P*	AUC	*P*
**Apical long-axis view**						
Apical posterior	0.56	0.38	0.51	0.90	0.59	0.19
Apical anteroseptal	0.56	0.41	0.51	0.86	0.58	0.25
Mid anteroseptal	0.56	0.38	0.53	0.60	0.62	0.06
Basal anteroseptal	0.55	0.46	0.57	0.18	0.59	0.17
**Apical 4-chamber view**						
Mid septal	0.52	0.74	0.51	0.80	0.63	0.05
Apical septal	0.54	0.54	0.51	0.86	0.70	< 0.01
Apical lateral	0.54	0.54	0.53	0.59	0.65	0.02
**Apical 2-chamber view**						
Apical inferior	0.68	< 0.01	0.53	0.63	0.62	0.06
Apical anterior	0.71	< 0.01	0.68	< 0.01	0.75	< 0.01
Mid anterior	0.67	< 0.01	0.60	0.12	0.59	0.19
Basal anterior	0.54	0.53	0.56	0.38	0.50	0.96

*P* values versus the line of random chance (AUC = 0.5).

AUC, area under the curve.

### Peak systolic strain, T_ESL_, and T_PSS_ in the apical anterior segment

**Figure 1:**
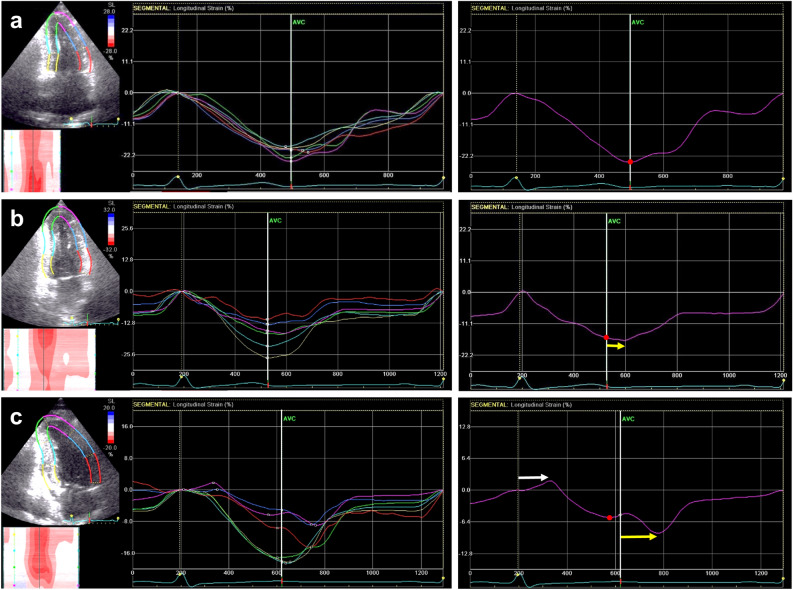
Representative strain–time curves in six segments (left) and the apical anterior segment (right) of the apical 2-chamber view. (**a**) An 83-year-old female with 29% diameter stenosis of the left anterior descending artery (LAD) derived from quantitative coronary angiography. (**b**) A 67-year-old male with 58% LAD stenosis. (**c**) A 56-year-old male with 88% LAD stenosis. The absolute value of the peak systolic strain (red circle) decreased, and early systolic lengthening (ESL) and postsystolic shortening (PSS) became larger with the severity of LAD stenosis, resulting in prolonged T_ESL_ (white arrow) and T_PSS_ (yellow arrow).

**Figure 2:**
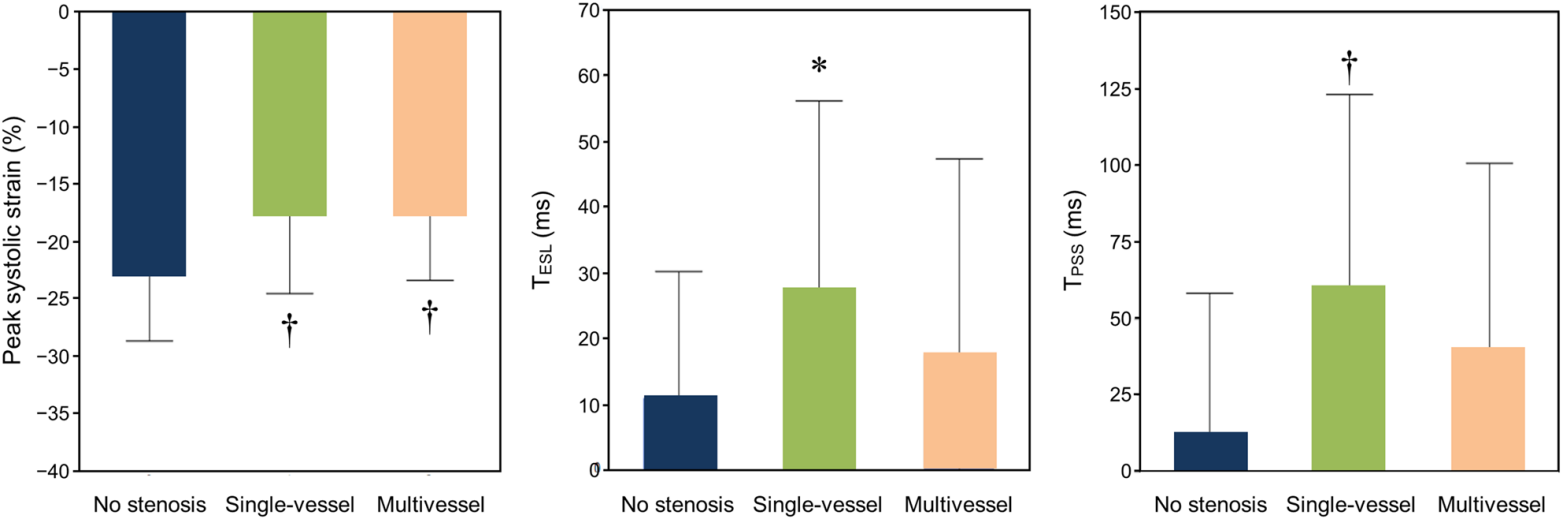
Peak systolic strain, T_ESL_, and T_PSS_ in the three groups. In the single-vessel group, the absolute value of the peak systolic strain was significantly lower, and T_ESL_ and T_PSS_ were significantly longer than those of the no stenosis group. In the multivessel group, the peak systolic strain was also significantly lower. T_ESL_ and T_PSS_ tended to be prolonged but not to a statistically significant degree. *P < 0.05 versus the no stenosis group. †*P* < 0.01 versus the no stenosis group.

Representative strain–time curves in the apical anterior segment are shown in Fig. 1. The absolute value of the peak systolic strain decreased, and ESL and PSS became larger with the severity of LAD stenosis, resulting in prolonged T_ESL_ and T_PSS_. In all data, the absolute value of the peak systolic strain was significantly lower, and T_ESL_ and T_PSS_ were significantly longer in the single-vessel group than in the no stenosis group. In the multivessel group, the peak systolic strain was also significantly lower. T_ESL_ and T_PSS_ tended to be longer but not to a statistically significant degree (Fig. 2).

### Integrated analysis of the three strain parameters

**Figure 3:**
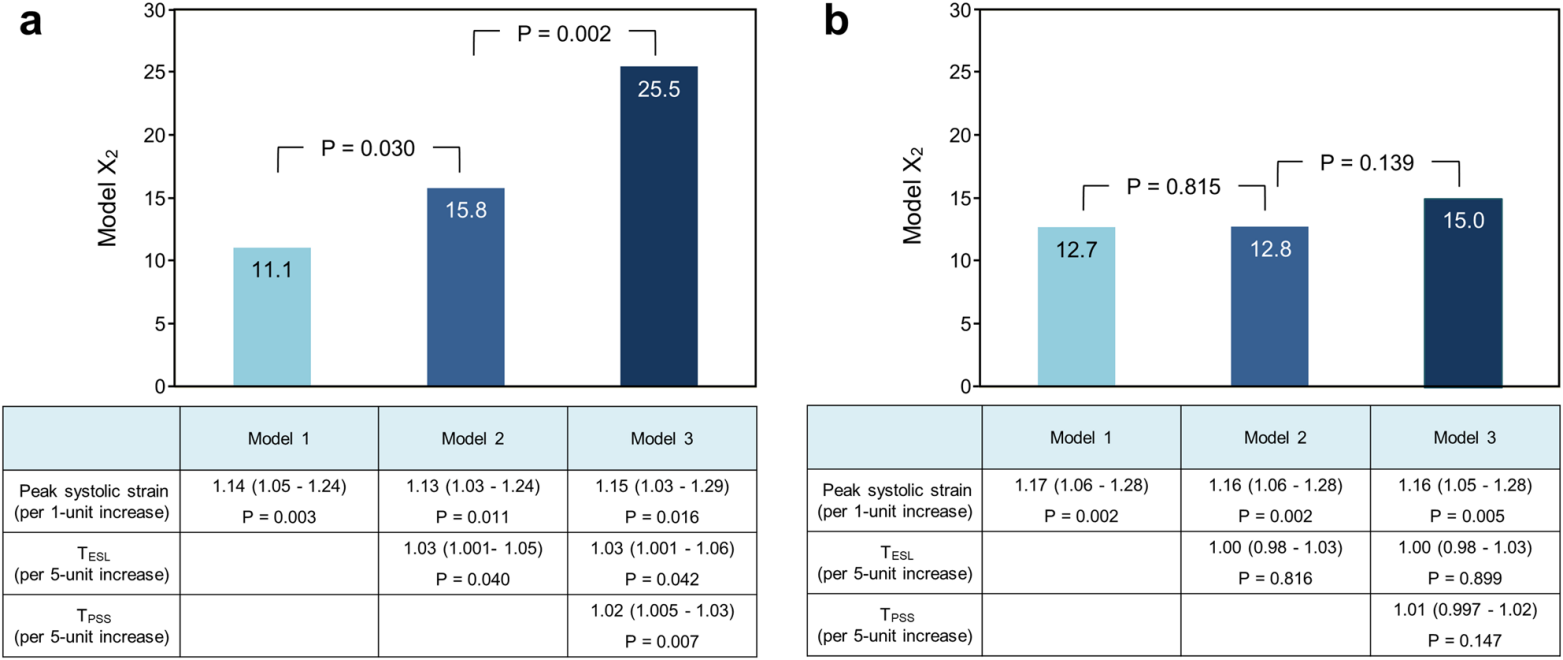
Incremental value of T_ESL_ and T_PSS_ by multivariable logistic regression models. (**a**) In the single-vessel group, the peak systolic strain was a significant parameter in diagnosing left anterior descending artery (LAD) stenosis, and T_ESL_ and T_PSS_ were also independent determinants in the logistic regression analysis. The addition of T_ESL_ and T_PSS_ to the peak systolic strain significantly increased the model power. (**b**) In the multivessel group, the peak systolic strain was also a significant parameter in diagnosing LAD stenosis. However, the addition of T_ESL_ and T_PSS_ did not generate a significant increase in the model power. The tables present the odds ratios and 95% confidence intervals.

In the single-vessel group, the peak systolic strain was a significant parameter for diagnosing LAD stenosis, and T_ESL_ and T_PSS_ were also independent determinants in the multivariable logistic regression analysis. The addition of T_ESL_ and T_PSS_ to the peak systolic strain significantly increased the model power, and the following regression function was acquired for the probability p to diagnose LAD stenosis: logit(p) = 1.59 + 0.14ε_S_ + 0.03T_ESL_ + 0.02T_PSS_, where ε_S_ = the peak systolic strain. In the multivessel group, however, the addition of T_ESL_ and T_PSS_ to the peak systolic strain did not generate a significant increase in the model power (Fig. 3).

**Figure 4:**
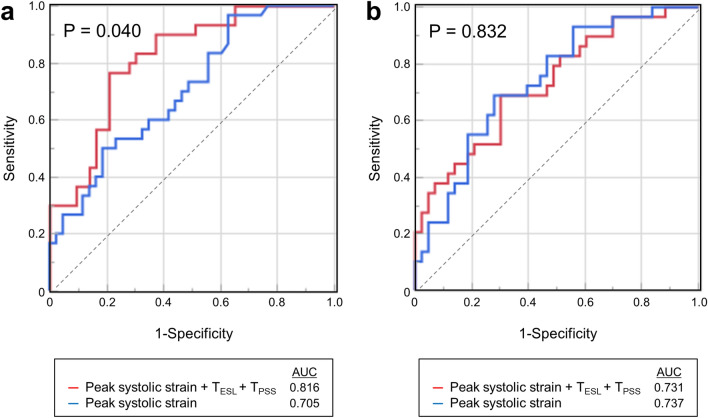
Receiver operating characteristic curve analysis for diagnosing left anterior descending artery (LAD) stenosis. (**a**) Compared with the peak systolic strain alone, the integrated analysis of the three parameters from using the regression function significantly improved the accuracy in diagnosing single-vessel LAD stenosis (*P* = 0.040). (**b**) The integrated analysis did not improve the accuracy in diagnosing multivessel LAD stenosis (*P* = 0.832).

The integrated analysis of the three parameters by using the abovementioned function significantly improved the accuracy in diagnosing single-vessel LAD stenosis (sensitivity of 77% and specificity of 79%) over the peak systolic strain alone. However, the integrated analysis did not improve the diagnostic accuracy in diagnosing multivessel LAD stenosis (Fig. 4).

### Follow-up echocardiography after revascularization

Follow-up echocardiography was evaluated in 16 patients with successful revascularization as confirmed by follow-up QCA (11 in the single-vessel group, 5 in the multivessel group). The duration between the first and follow-up echocardiography was 378 ± 229 days. Compared with those before revascularization, peak systolic strain and T_PSS_ were significantly improved after revascularization. T_ESL_ tended to be improved after revascularization but it was not significant due to large variation (Table 3).

**Table 3:** Echocardiographic parameters before and after revascularization (16 patients).

	Before revascularization	After revascularization	*P*
% diameter stenosis	63.3 ± 13.2	0.0 ± 0.0	< 0.01
LVEF (%)	64.1 ± 3.5	63.5 ± 5.2	0.61
GLS (%)	− 17.5 ± 1.7	− 18.2 ± 2.7	0.33
Peak systolic strain (%)	− 16.1 ± 5.7	− 22.8 ± 4.0	< 0.01
T_ESL_ (ms)	25.7 ± 32.8	20.4 ± 33.8	0.97
T_PSS_ (ms)	47.1 ± 55.3	6.0 ± 54.8	< 0.01

Data are presented as mean ± standard deviation.

GLS, global longitudinal strain; LVEF, left ventricular ejection fraction.

### Interobserver and intraobserver reliability

The intraclass correlation coefficients and Bland–Altman analyses of peak systolic strain, T_ESL_, and T_PSS_ are summarized in Tables 4 and 5, respectively. The intraclass correlation coefficients for both reliability levels were excellent for all myocardial strain parameters. The limits of agreement (± 1.96 standard deviation) of T_ESL_ and T_PSS_ in the Bland–Altman analyses were within ± 35 ms in the interobserver measurement and within ± 25 ms in the intraobserver measurement.

**Table 4:** Intraclass correlation coefficients (95% confidence intervals) in interobserver and intraobserver measurements for peak systolic strain, T_ESL_, and T_PSS_ in the apical anterior segment.

	Interobserver	Intraobserver
Peak systolic strain	0.85 (0.61–0.95)	0.86 (0.65–0.95)
T_ESL_	0.88 (0.69–0.96)	0.98 (0.95–0.99)
T_PSS_	0.92 (0.78–0.97)	0.96 (0.88–0.99)

**Table 5:** Mean differences and limits of agreement (± 1.96 standard deviation) in interobserver and intraobserver measurements for peak systolic strain, T_ESL_, and T_PSS_ in the apical anterior segment.

	Interobserver	Intraobserver
Peak systolic strain (%)	1.0 ± 8.4	− 1.8 ± 6.5
T_ESL_ (ms)	− 2.3 ± 32.0	− 2.3 ± 12.9
T_PSS_ (ms)	5.5 ± 34.1	1.6 ± 23.9

## Discussion

The main findings of this study are as follows: (1) The apical anterior segment in the 2-chamber view was the optimal segment for detecting single-vessel LAD stenosis by using nonstress speckle-tracking echocardiography. (2) In the apical anterior segment, the absolute value of the peak systolic strain decreased, and T_ESL_ and T_PSS_ were prolonged in the single-vessel group. (3) T_ESL_ and T_PSS_ were determinants independent of the peak systolic strain in detecting the single-vessel LAD stenosis. (4) The integrated analysis of the three parameters significantly improved the diagnostic accuracy of single-vessel LAD stenosis over that of the peak systolic strain alone, but it could not improve the diagnostic accuracy of multivessel LAD stenosis.

It is difficult to diagnose the presence of significant CAD in patients referred with suspected CAD using nonstress echocardiography because ischemia does not always occur in myocardium perfused by the culprit coronary artery. A stress test should be performed for such patients; however, it is often withheld in the acute phase of the ischemic episode as the patient may be in an unstable condition.

Myocardial strain analysis with tissue Doppler or speckle-tracking echocardiography can not only quantify regional myocardial function but also detect subtle myocardial deformations, such as ESL or PSS, which are difficult to detect with conventional echocardiography^2,3^. ESL and PSS occur in myocardium with contractile dysfunction through an imbalance of tension between the damaged myocardium and the surrounding normal (or less damaged) myocardium^14,15^ and become larger with the severity of myocardial ischemia^16,17^. Each assessment of ESL or PSS allows the sensitive detection of significant CAD in the clinical setting^4–9^. Moreover, it has been reported that ESL and/or PSS allow assessing myocardial ischemic memory because they persist after transient ischemia in animal studies^18–20^. Therefore, we hypothesized that in some patients referred with suspected CAD, ESL and PSS might appear in the ischemic myocardium even at rest due to silent ischemia or ischemic memory. In the present study, the decrease in the peak systolic strain and the prolongation of T_ESL_ and T_PSS_ were demonstrated in the single-vessel group despite the use of nonstress echocardiography, which seems to support our hypothesis.

ESL and PSS can be observed in the normal myocardium^10^. Since the presence of myocardial ischemia at rest could not be indicated in each patient, it is unclear whether the ESL and PSS observed in this study reflect myocardial ischemia. However, compared with those before revascularization the peak systolic strain and T_PSS_ were significantly improved, and T_ESL_ tended to be improved after revascularization. This recovery suggests that the decrease in peak systolic strain and the prolongation of T_ESL_ and T_PSS_ were caused by ischemia.

In the detection of single-vessel LAD stenosis, although the AUCs of T_ESL_ and T_PSS_ in the apical anterior segment were significantly larger than the line of random chance, as shown in Table 2, they were not significantly different from the AUC of the peak systolic strain. These results imply that the assessment of T_ESL_ or T_PSS_ alone could not improve the diagnostic accuracy over that of the peak systolic strain in our cohort.

To date, it had not been fully ascertained whether ESL and PSS are determinants independent of the peak systolic strain in diagnosing CAD. In this study, we proved that T_ESL_ and T_PSS_ were independent determinants in the differentiation of the single-vessel group from the no stenosis group. Furthermore, the integrated analysis of the peak systolic strain, T_ESL_, and T_PSS_ improved the diagnostic accuracy in detecting single-vessel LAD stenosis. These results suggest that the integrated analysis of the three parameters is useful for better screening single-vessel LAD stenosis by using nonstress echocardiography.

In multivessel LAD stenosis, although T_ESL_ and T_PSS_ tended to be prolonged, they were not determinants independent of the peak systolic strain, and the integrated analysis could not improve the diagnostic accuracy. As mentioned above, ESL and PSS occur through an imbalance of tension between the damaged and surrounding myocardium. In the multivessel group, LVEF was preserved, but the GLS was significantly smaller than that of the no stenosis group. Dysfunction in the myocardium perfused by RCA and/or LCX may affect the occurrence of ESL and PSS in the LAD territory.

The frame rate strongly influences the assessment of T_ESL_ and T_PSS_. Since it was set at 52– 84 frames/s in the present study, the time interval between frames was 11.9–19.2 ms. Although ESL and PSS shorter than this duration may not have been detectable, we think that the temporal resolution was enough to detect significant ESL and PSS.

Our study has limitations that are described below. Although the integrated analysis of the three parameters improved the diagnostic accuracy, whether the regression function used in this study could be adapted to other similar cohorts was not assessed. The usefulness of the integrated analysis in detecting RCA and LCX stenosis must also be tested in further studies. In addition, the use of the regression function may be complicated in the clinical setting. A simpler parameter that reflects ESL and PSS should be devised.

The apical anterior segment was selected as optimal for detecting LAD stenosis. We initially expected that the apical septal segment in the apical 4-chamber view would show similar diagnostic accuracy, but the diagnostic accuracy in the apical septal segment was lower than that in the apical anterior segment. Although the reason for this is unclear, some patients’ true apex may not have been able to be included due to the foreshortened 4-chamber view. Analysis in multiple segments should be done in future investigations.

As mentioned above, the presence or absence of myocardial ischemia at rest was not confirmed for each patient. The integrated analysis demonstrated a high diagnostic accuracy in this study; however, false negatives can be present as long as nonstress echocardiography is performed. We therefore recommend that integrated analysis be used as a screening test for patients with suspected CAD but without visual wall motion abnormalities. In the apical anterior segment, a decrease in the peak systolic strain suggests LAD stenosis, including multivessel LAD stenosis, but if the peak systolic strain is normal, we should add an integrated analysis to increase the diagnostic accuracy in patients with single-vessel LAD stenosis.

## Conclusions

ESL and PSS were determinants independent of the peak systolic strain in detecting single-vessel LAD stenosis by using nonstress speckle-tracking echocardiography, and compared with the peak systolic strain alone, the integrated analysis of the peak systolic strain, T_ESL_, and T_PSS_ significantly improved the diagnostic accuracy of single-vessel stenosis. In contrast, the incremental diagnostic value of T_ESL_ and T_PSS_ could not be demonstrated in multivessel stenosis.

## Data Availability

The datasets generated during and/or analyzed during the current study are available from the corresponding author on reasonable request.
